# Relationship between active *Helicobacter pylori* infection and risk factors of cardiovascular diseases, a cross-sectional hospital-based study in a Sub-Saharan setting

**DOI:** 10.1186/s12879-022-07718-3

**Published:** 2022-09-12

**Authors:** Lionel Danny Nguefak Tali, Ghislaine Florice Nintewoue Faujo, Justine Laure Nguieguia Konang, Jean Paul Dzoyem, Laure Brigitte Mabeku Kouitcheu

**Affiliations:** 1 Microbiology and Pharmacology Laboratory, Department of Biochemistry, Faculty of Science, University of Dschang, P.O. Box 67, Dschang, Cameroon; 2 Regional Hospital Bafoussam, P. O. Box 980, Bafoussam, Cameroon; 3 Medical Microbiology Laboratory, Department of Microbiology, Faculty of Science, University of Yaoundé I, P.O. Box 812, Yaoundé, Cameroon

**Keywords:** Dyslipidemia, Hypertension, Overweight/obesity, Cardiovascular risk factors, *Helicobacter pylori* infection, Cameroon

## Abstract

**Background:**

Chronic inflammation has been reported as one of the novel coronary heart disease (CHD) risk factors. Knowing that *Helicobacter pylori* (*H. pylori*) provokes a local inflammation, the relationship between *H. pylori* infection and cardiovascular disease (CVD) has received considerable attention. However, the attempt to demonstrate the association between *H. pylori* and specific cardiovascular disease risk factors is always a challenging issue due to the conflicting reports in the literatures.

**Methods:**

We performed a cross-sectional study of 363 consecutive dyspeptic subjects in three reference health facilities in Cameroon from October 2020 to October 2021. Each participation gave a written consent and the study was approved by the local Ethical Committee. Check-up for cardiovascular disease (CVD) risk factors such as dyslipidemia-related parameters, obesity-related parameter, high blood pressure as well as *H. pylori* detection was done for each participant. Data was analyzed using SSPS statistical package.

**Results:**

*Helicobacter pylori* infection was significantly associated with higher total cholesterol level (OR: 2.3324, p = 0.0002) and higher LDL cholesterol level (OR: 2.3096, p = 0.0006). The crude OR of *H. pylori* status on the prevalence of high body mass index (BMI) was 1.0813 (p = 0.7300) and the adjusted OR for confounding factors was 1.1785 (p = 0.5095). The strength of the association between *H. pylori* infection and blood pressure, shows an OR of 1.3807 (p = 0.2991), 1.0060 (p = 0.9855) and 1.4646 (p = 0.2694) for diastolic pressure, hypertension and high heart rate respectively, while that of systolic pressure was 0.8135 (p = 0.4952). *H. pylori* infection is associated with dyslipidemia in our milieu.

## Background

Cardiovascular diseases (CVD) involves coronary heart disease or cerebrovascular disease [1]. They are the primary cause of death in the Western world [1]. Coronary heart disease is responsible for nearly 735,000 heart attacks [2]. It is estimated that 630,000 of people die for coronary disease each year in the U.S [2]. Cerebrovascular disease is related to atherothrombosis mechanism [1], whereas coronary heart disease occurs when the lumen of arteries that supply the heart has been reduced as a result of plaque builds up in their walls [2]. Because heart disease is widely found and sometimes progress to an ultimate and complex state without precursor’s signs, it is important to identify their associated risk factors. The more someone is exposed to these risk factors, the greater is its probability to acquire coronary heart disease (CHD) and heart attack. The American Heart Association recommends that attention should be pay in prevention of heart disease early in life. The sooner you assess and manage your risk factors, the higher your opportunity to live without heart attack.

Several risk factors of coronary heart disease and heart attack has been identified and reported in the literature [3]. These included high blood pressure (BP), high blood cholesterol levels, smoking, alcohol consumption, diabetes, overweight or obesity, lack of physical activity, unhealthy diet and stress known as modifiable traditional risk factors; getting older, male sex, being postmenopausal, family history of heart disease, and race which cannot be controlled. However, a great percentage of patients with CVD are without these traditional risks [4], suggesting that other factors may contribute in the development of this chronic disease. Chronic inflammation has been reported as one of the novel coronary heart disease (CHD) risk factors [5]. Chronic infection in general and undetected ones particularly has been incriminated as the cause of these inflammatory condition, emphasizing the implication of microorganisms that are commonly detectable in asymptomatic individuals in this chronic process [6]. The proposed mechanism by which these pathogen may lead to CVD included direct spoil of the vessel wall with local vascular inflammation as consequence [7], or indirect induction of endothelial dysfunction and dyslipidemia, resulting in CVD through systemic inflammation [8]. *Helicobacter pylori* is a Gram-negative bacterium that infects the gastric mucosa [9]. With nearly half of the world’s population been affected, *Helicobacter pylori* infection represents a significant health burden [10]. Despite the fact that a high proportion of infected individuals remains asymptomatic or displays rather minor unspecific symptoms [11], infection with this pathogen may lead to gastro-duodenal pathologies which ranges from mild gastritis to gastric adenocarcinoma [12] and have been associated in the pathogenesis of some extra gastrointestinal disorders [13, 14]. Mucosal damage induced by gastric *Helicobacter pylori* infection result both from the direct effects of bacterial virulence factors and as a consequence of the inflammatory response elicited by the bacterium [15]. Derya et al. in their study noticed that gastric mucosal IFN-ϒ, TNF-α, IL-6, IL-10, IL-17A mRNA genomic expressions were increased in *H pylori*-infected chronic active gastritis compared with normal gastric mucosa groups [16]. The authors also noticed that the relative mRNA expressions of TNF-α, IL-6, IL-10, IL-17A, TGF-β were found to have a significant increase between gastric carcinoma and control patients [16]. *H. pylori*-specific gastric mucosal T cell responses are Th1 and Th17, with Th1 in the greatest amount [17, 18]. Th17 has demonstrated a significant contribution to the progression of autoimmune disease and inflammation in the gastrointestinal tract [19]. IL-17 is the main cytokine from Th17 cell and has many associated cytokines, such as IL-1β, IL-6, IL-21, and IL-23 [19]. IL-17 together with IL-21 and IL-23 plays a role in inflammation site formation, either directly or indirectly. The IL-17/IL-21 and IL-17/IL-23 axes have multiple inflammatory effect on epithelial, endothelial, and fibroblast cells [20, 21]. IL-21 increases proinflammatory cytokines released from macrophages, enhancing proliferation of lymphoid cells, and promoting B cell differentiation, whereas the expression of IL-23 in patients with *H. pylori* infection is elevated and positively correlates with the degree of neutrophils and monocyte infiltration [22].

Knowing that the vast majority of *H. pylori* infected individuals remains asymptomatic, that *Helicobacter pylori* provokes a local inflammation in almost all host [12], that this pathogen is implicated in extra digestive disorders, and that local and systemic inflammation by microbes and infectious agents is a cardiovascular disease risk factor [13, 14], the relationship between *H. pylori* infection and CVD has received considerable attention [23, 24].

However, results from the studies on the relationship between *H. pylori* infection and CVD are quite controversial. Some reports demonstrated a substantial higher prevalence of *H. pylori* infection in patients with previous history of CVD and supported a possible link between *H. pylori* infection and CVD development [25, 26]. In contrast, other reports showed that the seroprevalence of *H. pylori* was not associated with coronary artery disease and that *H. pylori* status did not determine the risk of CVD [27, 28].

If *H. pylori* is a possible contributor to cardiovascular disease (CVD) progression, this pathogen might directly or indirectly induces endothelial dysfunction leading to vascular inflammation and atherosclerosis or this pathogen by acting on traditional risk factors of CVD might probably induce atherosclerosis progression. Hence, earlier diagnosis and possibly eradication of *H. pylori* might be necessary for preventing atherosclerosis progression, especially in high-risk population for *H. pylori* infection. In Cameroon, *H. pylori* infection rate ranging from 92.2 to 64.34% respectively in the North-west and the Littoral Regions [29, 30], which is higher than the infection rate of 34.1% reported in Thailand [31]. On the other hand, non-communicable diseases are estimated to account for 31% of all deaths in the country, 14% of which are due to cardiovascular diseases [32]. Thus, it is important to identify and manage factors affecting cardiovascular risk in order to live without heart attack. In this study, it is assumed that the association between *H. pylori* and cardiovascular disease may be behind the impact of *H. pylori* on traditional risk factors of CVD. Hence, this study was aimed to assess the association between *H. pylori* infection and some individual traditional risk factors of cardiovascular disease or atherosclerosis progression including, high blood pressure or hypertension, dyslipidemia, and high body mass index among dyspeptic population in Cameroon. The impact of socio-economic factors, smoking, alcohol consumption, lack of physical activity, family history of heart disease was also evaluated on the outcome of these CVD risk factors.

## Subjects and methods

### Study participants

This study included 363 subjects complaining for gastrointestinal disorders and who have undergone upper endoscopy with *H. pylori* detection using histological examination, and check-up for cardiovascular disease (CVD) risk factors such as high body mass index, high blood pressure, dyslipidemia at the Regional Hospital Bafoussam, General Hospital Yaoundé and Cathedral Medical Centre Yaoundé from October 2020 to October 2021. We employed a consecutive sampling for data collection, requesting consent from all volunteer patients in the selected health facilities who fulfilled the eligibility criteria for the study during the study period. To avoid confounding bias, we excluded possible confounding medical conditions known to be associated with the health outcome. The exclusion criteria were as follows: (1) patients with any gastrointestinal medications (Antibiotics for *H. pylori* eradication or proton pump inhibitors); (2) patients who frequently use non-steroidal anti-inflammatory drugs; (4) Pregnant and breastfeeding women; (4) patients under 15 years of age and (5) patients for whom endoscopic and blood sampling was not possible. Non-cooperative patients were also excluded.

### Assessment of cardiovascular risk factors

#### Body mass index (BMI) determination

For each participant, height and weight were measured and used to calculated body mass index (BMI) as follows: BMI (kg/m^2^): [Weigh (kg)]/[Height (m)]^2^. According to BMI value, participants were classified in 3 categories; normal (< 25 kg/m^2^), overweight (25 to 30 kg/m^2^) and obese (> 30 kg/m^2^) [33].

#### Assessment of heart dysfunction

Heart dysfunction was evaluated through blood pressure and heart rate measurement following the World Health Organization (WHO) guidelines [34]. These parameters were taken for each participant by trained technicians after resting for at least 5 min in a sitting position using an Omron^®^ digital memory blood pressure monitor (Health Care Co Ltd, Kyoto, Japan). Hypertension was defined according to WHO/International Society of Hypertension guidelines as systolic blood pressure (SBP) ≥ 140 mmHg and/or diastolic blood pressure (DBP) ≥ 90 mmHg [34]. Eligible subjects previously aware for their hypertension status were also considered as having hypertension. Patients were classified relative to heart rate into three groups; those having tachycardia (heart rate < 60 beats/min), those having bradycardia (heart rate > 100 beats/mn) and those with normal heart rate (60 to 100 beats/min).

#### Assessment of dyslipidemia

About 5 ml of venous blood were collected in a dry tube from each subject and were used to determine the serum levels of total cholesterol, high-density lipoprotein cholesterol (HDL-C), low-density lipoprotein cholesterol (LDL-C) and triglyceride (TG) using colorimetric methods (Biosystem Costa brava, 3008030, Spain). The sensitivity of each test as detection limit was 4.4 mg/dl or 0.05 mmol/l for TG, 0.28 mg/dl or 0.007 mmol/l for LDL-C, 0.5 mg/dl or 0.01 mmol/l for HDL-C and 0.3 mg/dl or 0.008 mmol/l for TC. Total cholesterol value > 200 mg/dl was defined as hypercholesterolemia, LDL-cholesterol value > 130 mg/dl as high LDL cholesterol, triglycerides value > 150 mg/dl as hyper triglyceridaemia, and HDL-cholesterol value < 40 mg/dl as low HDL cholesterol.

#### Assessment of *H. pylori* infection

During Esophagogastroduodenoscopy examinations (FOGD), biopsy samples were collected from the antrum, the fundus and the angulus for *H. pylori* detection. These specimens, were fixed in formalin 10% and send to anatomo-pathology laboratory for histologic analysis. Gastric biopsies were removed from formalin and inserted into an accessioned cassette. The cassette was placed into a tissue processing automate for dehydration, clearing and impregnation with liquid paraffin. The biopsy obtained at the end of this tissue processing step was embedded in paraffin, cut into fine sections using a microtome and then placed on glass slides. The slides were stained using Giemsa-stained for *H. pylori* detection. The results were scored blindly with the use of patients codes.

#### Assessment of others variables

Information on sociodemographic and economic factors (age, sex, and income level), lifestyle (smoking, alcohol consumption, and physical activity), medication history (current and regular use of antibiotics, proton pump inhibitors (PPI) consumption, non-steroidal anti-inflammatory drugs (NSAIDs) consumption, antihypertensive medication), and personal medical history (hypertension status, diabetic status, family history of hypertension, family history of diabetes mellitus, and family history of obesity) were requested from the subjects in a semi-structured questionnaire.

Socioeconomic class or income level was stratified into low income (≤ 2500$/month), middle income (2500–8500$/month) and high income (≥ 8500$/month) [10].

Regular exercise was defined as physical activity at least 30 min ≥ 3 days per week [35]. Smoking status was assessed as never smoker, former smoker, or current smoker. Current smoking was defined as having smoked at least one cigarette per day for more than half a year. Those who had smoked but stopped smoking for more than 1 month during the survey were defined as former smoking [35].

Current alcohol consumption referred to those who were drinking at least one time per week for more than half a year, former drinker referred to those who had previously consumed alcohol but stopped drinking for more than 6 months and never or occasionally (once or twice per month) [35].

### Statistical analysis

Results from were expressed as means ± standard deviation, ratio or percentages. Student t-test were used to compare groups of continuous variables, the Fisher exact test or Chi-square test for categorical variables. The strength of the association between *H. pylori* status and risk factor of cardiovascular diseases was assessed by means of odd risks (ORs) with 95% confidence intervals (CIs) using univariable and multivariable logistic regression analyses. The variables used for the multivariable analysis included; sociodemographic and economic factors (age, sex, income level), life style (smoking status, alcohol consumption, physical activity), and personal medical histories (history of hypertension, history of diabetes mellitus, history of obesity) stratified respectively as model 1, 2, 3 multivariate logistic analysis and model 4 for the overall variable. A p-value < 0.05 was considered statistically significant. SPSS statistical package (Windows version 19.0) was used for analysis.

## Results

### Characteristics of the study population

A total of 363 consecutive dyspeptic subjects who underwent upper endoscopy in the Gastroenterology Department of the selected health facilities were included in this study. Out of the 363 subjects, 188 (51.79%) were women and 175 (48.21%) were men, their mean age was 47.53 ± 17.07 years and ranged from 15 to 97 years old. Participants were subdivided into five age group (≤ 20, [20–35], [35–50], [50–65] and > 65 years old) and the age group from 50 to 65 years the most represented (33.66%; 122/363). Fifty two percent of subjects (52.28%, 192/363) were from middle class with 2500–8500$/month, followed by 46.00% (167/363) from low income or poorly skilled class and 1.10% (4/363) from elite class with relatively high income (≥ 8500$/month). Regarding smoking and alcohol consumption, participant were classified into non-smokers and non-drinkers because the number of former smokers or former drinkers were not representative in our sample population. Current smokers and drinkers frequency were 5.5% (20/363) and 23.41% (85/363) respectively in our population. Regular exercise was recorded in 42.42% (154/363) of subjects and among the 363 participants, 17.63% (64), 25.06% (91) and 16.80% (61) had a family history of hypertension, diabetes mellitus and obesity respectively (Table 1).

**Table 1:** Characteristics of the study population

Variables	Number (%)
Socio-demographic and economic	
Sex. ratio (1/1.07)	
Female	188 (51.79)
Male	175 (48.20)
Age (years). Mean ± SD. [min–max]	47.53 ± 17.07 [15–97]
≤ 20	22 (6.06)
[20–35]	72 (19.83)
[35–50]	101 (27.82)
[50–65]	122 (33.66)
> 65	46 (12.67)
Income level ($/month)	
Unemployed or low class (≤ 2500)	167 (46.00)
Middle class (2500–8500)	192 (52.89)
Elite (≥ 8500)	4 (1.10)
Lifestyle	
Smoking	
Yes	20 (5.5)
No	343 (94.5)
Alcohol consumption	
Yes	85 (23.41)
No	278 (76.58)
Physical activity	
Yes	154 (42.42)
No	209 (57.58)
Medical history	
History of hypertension	
Yes	64 (17.63)
No	282 (77.68)
History of diabetes mellitus	
Yes	91 (25.06)
No	253 (69.69)
History of obesity	
Yes	61 (16.80)
No	284 (78.23)

### Distribution of cardiovascular risk factors in the study population

As far as risk of dyslipidemia-related variables are concerned, the mean value of total cholesterol, LDL cholesterol, HDL cholesterol and triglycerides were 215.38 ± 59.58 g/l (range 86–412 g/l), 110.02 ± 45.21 g/l (12.98–229.6 g/l), 51.79 ± 21.03 g/l (4.3–119 g/l) and 145.91 ± 61.94 (13.39–318) respectively among our sample population. Hypercholesterolemia (> 200 g/l) was detected in 61.70% (224/363), high LDL cholesterol (> 130 g/l) in 37.19% (135/363), low HDL cholesterol (< 40 g/l) in 39.12% (142/363) and hyper-triglyceridemia in 44.07% (160/363) of the overall subjects. Regarding dyslipidemia in relation to socio-demographic, lifestyle and medical history of participants, individuals with family history of hypertension (OR: 2.162 (1.247–3.798), p = 0.0059) and those aged above 35 years old (X^2^ = 12.9720, p = 0.0114) were more prone to hypercholesterolemia, those with middle or high level income were more prone to high LDL cholesterol (OR: 1.7401 (1.1330–2.6727), p = 0.0114) and men were more prone to low HLD cholesterol than women (OR: 1.6307 (1.0638–2.4956), p = 0.0248), (Table 2).

**Table 2:** Distribution of dyslipidemia-related variables according to socio-economic, lifestyle and medical history factors

Variable	N	Present n (%)	Absent n (%)	OR (95% CI) [X^2^]	p value
Hyper total cholesterol		224	139		
Age (years)					
≤ 20	22	12 (54.54)	10 (45.46)	[12.9720]	0.0114*
[20–35]	72	33 (45.83)	39 (54.17)		
[35–50]	101	63 (62.38)	38 (37.62)		
[50–65]	122	87 (73.31)	35 (26.69)		
> 65	46	29 (63.03)	17 (36.97)		
Age ≤ 20					
Yes	22	12 (54.55)	10 (45.45)	1.3695 (0.5753–3.2600	0.4773
No	341	212 (62.17)	129 (37.83)		
Gender					
Female	188	118 (62.77)	70 (37.23)	0.9113 (0.5967–1.3918)	0.6673
Male	175	106 (60.57)	69 (39.43)		
Income level					
Low	167	105 (62.87)	62 (31.12)	[2.3822]	0.3039
Middle	192	118 (56.25)	74 (38.54)		
High	4	1 (25.00)	3 (75.00)		
Low income level					
Yes	152	95 (62.50)	57 (37.50)	0.8730 (0.5747–1.3263)	0.5245
No	196	119 (60.71)	77 (39.29)		
Alcohol consumption					
Yes	85	46 (54.12)	39 (45.88)	0.6626 (0.4052–1.0837)	0.1011
No	278	178 (64.03)	100 (35.97)		
Physical activity					
Yes	154	92 (59.74)	62 (40.26)	0.8656 (0.5645–1.3272)	0.5081
No	209	132 (63.16)	77 (36.84)		
History of hypertension					
Yes	64	34 (53.13)	30 (46.88)	2.162 (1.247–3.798)	0.0059*
No	282	97 (34.40)	185 (65.60)		
History of diabetes mellitus					
Yes	91	58 (63.74)	33 (36.26)	1.0744 (0.6535–1.7662)	0.7773
No	253	157 (62.06)	96 (37.94)		
History of obesity					
Yes	61	44 (72.13)	17 (27.87)	1.6851 (0.9172–3.0956)	0.0926
No	284	172 (60.56)	112 (39.44)		
High LDL-C		135	228		
Age (years)					
≤ 20	22	14 (63.63)	8 (36.36)	[2.9524]	0.5658
[20–35]	72	49 (68.05)	23 (31.94)		
[35–50]	101	62 (61.38)	39 (38.61)		
[50–65]	122	71 (58.20)	51 (41.81)		
> 65	46	32 (69.57)	14 (30.43)		
Age ≤ 20					
Yes	22	8 (36.36)	14 (63.64)	1.0384 (0.4239–2.5437)	0.9343
No	341	127 (37.24)	214 (62.76)		
Gender					
Female	188	72 (38.30**)**	116 (61.70**)**	0.9063 (0.5918–1.3881)	0.6511
Male	175	63 (36.00)	112 (64.00)		
Income level					
Low	167	52 (31.14)	115 (68.86)	[6.7140]	0.0348*
Middle	192	80 (41.66)	112 (58.34)		
High	4	3 (75.00)	1 (25.00)		
Low income level					
Yes	152	46 (30.26)	106 (69.74)	1.7401 (1.1330–2.6727)	0.0114*
No	196	83 (42.35)	113 (57.65)		
Smoking					
Yes	20	7(35.00)	13 (65.00)	0.9055 (0.3522–2.3283	0.8368
No	343	128 (37.32)	215 (62.68)		
Alcohol consumption					
Yes	85	31 (36.47)	54 (63.53)	0.9606 (0.5803–1.5901)	0.8757
No	278	104 (37.41)	174 (62.59)		
Physical activity					
Yes	154	55 (35.71)	99 (64.29)	0.8961 (0.5819–1.3799)	0.6184
No	209	80 (38.28)	129 (61.72)		
History of hypertension					
Yes	64	21 (32.81)	43 (67.19)	0.7413 (0.4177–1.3157)	0.3064
No	282	112 (39.72)	170 (60.28)		
History of diabetes mellitus					
Yes	91	35 (38.46)	56 (61.54)	0.9885 (0.6042–1.6173)	0.9634
No	253	98 (38.74)	155 (61.26)		
History of obesity					
Yes	61	28 (45.90)	33 (54.10)	1.4248 (0.8155–2.4895)	0.2137
No	284	106 (37.32)	178 (62.68)		
Low HDL-C		142	221		
Age (years)					
≤ 20	22	9 (40.91)	13 (59.10)	[0.3881]	0.9834
[20–35]	72	27 (37.50)	45 (62.5)		
[35–50]	101	39 (38.63)	62 (61.37)		
[50–65]	122	50 (40.98)	72 (59.02)		
> 65	46	17 (36.96)	29 (63.04)		
Age ≤ 20					
Yes	22	9 (40.91)	13 (59.09)	0.9231 (0.3839–2.2194)	0.8581
No	341	133 (39.00)	208 (61.00)		
Gender					
Male	175	58 (33.14)	117 (66.86)	1.6307 (1.0638–2.4956)	0.0248*
Female	188	84 (44.68)	104 (55.32)		
Income level					
Low	167	63 (37.72)	104 (62.27)	[0.6540]	0.7211
Middle	192	78 (40.62)	114 (59.37)		
High	4	1 (25.00)	3 (75.00)		
Low income level					
Yes	152	56 (36.84)	96 (63.16)	1.1154 (0.7348–1.6932)	0.6079
No	196	79 (40.31)	117 (59.69)		
Smoking					
Yes	20	7 (35.00)	13 (65.00)	0.8296 (0.3228–2.1324)	0.6982
No	343	135 (39.36)	208 (60.64)		
Alcohol consumption					
Yes	85	31 (36.47)	54 (63.53)	0.8637 (0.5225–1.4278)	0.5678
No	278	111 (39.93)	167 (60.07)		
Physical activity					
Yes	154	58 (37.66)	96 (62.34)	0.8993 (0.5865–1.3788)	0.6263
No	209	84 (40.19)	125 (59.81)		
History of hypertension					
Yes	64	26 (40.63)	38 (59.38)	1.0864 (0.6247–1.8895)	0.7691
No	282	109 (38.65)	173 (61.35)		
History of diabetes mellitus					
Yes	91	31 (34.07)	60 (65.93)	0.7649 (0.4634–1.2624)	0.2944
No	253	102 (40.32)	151 (59.68)		
History of obesity					
Yes	61	20 (32.79)	41 (67.21)	0.7274 (0.4053–1.3056)	0.2862
No	284	114 (40.14)	170 (59.86)		
High TG		160	203		
Age (years)					
≤ 20	22	9 (40.91)	13 (59.09)	[4.3294]	0.3633
[20–35]	72	26 (36.13)	46 (63.87)		
[35–50]	101	42 (41.59)	59 (58.41)		
[50–65]	122	59 (48.36)	63 (51.64)		
> 65	46	24 (52.17)	22 (47.83)		
Age ≤ 20					
Yes	22	9 (5.56)	13 (6.40)	1.1466 (0.4774–2.7541)	0.7596
No	341	151 (94.37)	190 (93.56)		
Gender					
Female	188	81 (43.09)	107 (56.91)	1.0871 (0.7181–1.6457)	0.6932
Male	175	79 (45.14)	96 (54.86)		
Income level					
Low	167	68 (40.72)	99 (59.28)	[1.4309]	0.4890
Middle	192	90 (46.87)	102 (53.13)		
High	4	2 (50.00)	2 (50..00)		
Low income level					
Yes	152	63 (41.45)	89 (58.55)	1.2392 (0.8224–1.8671)	0.3053
No	196	92 (46.94)	104 (53.06)		
Smoking					
Yes	20	12 (60.00)	8 (40.00)	1.9764 (0.7878–4.9580)	0.1466
No	343	148 (43.15)	195 (56.85)		
Alcohol consumption					
Yes	85	42 (49.41)	43 (50.59)	1.3244 (0.8136–2.1558)	0.2584
No	278	118 (42.45)	160 (57.55)		
Physical activity					
Yes	154	69 (44.81)	85 (55.19)	1.0526 (0.6923–1.6006)	0.8104
No	209	91 (43.54)	118 (56.46)		
History of hypertension					
Yes	64	31 (48.44)	33 (51.56)	1.1979 (0.6955–2.0633)	0.5150
No	282	124 (43.97)	158 (56.03)		
History of diabetes mellitus					
Yes	91	44 (48.35)	47 (51.65)	1.1983 (0.7413–1.9371)	0.4604
No	253	111 (43.87)	142 (56.13)		
History of obesity					
Yes	61	25 (40.98)	36 (59.02)	0.8111 (0.4628–1.4214)	0.4645
No	284	131 (46.13)	153 (53.87)		

LDL-C: Low-density lipoprotein cholesterol; HDL-C: High density lipoprotein cholesterol; TG: Triglycerides; N, n: Number; X^2^: Chi square; (95% CI): 95% confidence intervals; OR: Odd ratio; Bold value with *: Significant., Chi-square value are in []

Approximately 13% (12.94%, 47/363) of participants were with high blood pressure or hypertension (blood pressure > 140/90 mmHg), 16.52% (60) and 15.15% (55) respectively with high diastolic blood pressure (> 90 mmHg) and systolic blood pressure (> 140/90 mmHg). High heart rate (> 100 beat/min) was detected in 13.22% (48/363) of patients (Table 3). The mean value of diastolic blood pressure, systolic blood pressure and heart rate was 77.024 ± 11.21 mmHg (range: 41–104 mmHg), 124.11 ± 19.29 mmHg (range: 80–191 mmHg) and 79.53 ± 12.10 beat/min (range: 45–114 beat/min) respectively. None of the evaluated sociodemographic, lifestyle and medical history parameters have a significant impact on blood pressure. However, individuals with history of obesity were with a high risk to have high heart rate than unexposed ones (OR: 2.5221 (1.2442–5.1124), p = 0.0103), (Table 3).

**Table 3:** Distribution of high blood pressure and high heart rate according to socio-economic, lifestyle and medical history factors

Variable	N	Present n(%)	Absent n(%)	OR (95% CI)	p value
High diastolic pressure		60	303		
Age (years)					
≤ 20	22	4 (18.18)	18 (81.82)	[9.0621]	0.0596
[20–35]	72	5 (6.94)	67 (93.06)		
[35–50]	101	19 (18.81)	82 (81.19)		
[50–65]	122	27 (22.13)	95 (77.87)		
> 65	46	5 (10.87)	41 (89.13)		
Age ≤ 20					
Yes	22	4 (18.18)	18 (81.82)	0.8842 (0.2883–2.7117)	0.8296
No	341	56 (16.42)	285 (83.58)		
Gender					
Female	188	31 (16.49)	157 (83.51)	1.0060 (0.5779–1.7510)	0.9832
Male	175	29 (16.57)	146 (83.43)		
Income level					
Low	167	31 (18.56)	136 (81.44)	[1.5753]	0.4549
Middle	192	29 (15.11)	163 (84.89)		
High	4	0 (0.00)	4 (100.00)		
Low income level					
Yes	152	28 (18.42)	124 (81.58)	0.7443 (0.4290–1.2912)	0.2934
No	196	29 (14.80)	167 (85.20)		
Smoking					
Yes	20	5(25.00)	15(75.00)	1.7490 (0.6109–5.0077)	0.2976
No	343	55 (16.03)	288 (83.97)		
Alcohol consumption					
Yes	85	17 (20.00)	68 (80.00)	1.3664 (0.7329–2.5477)	0.3260
No	278	43 (15.47)	235 (84.53)		
Physical activity					
Yes	154	23 (14.94)	131 (85.06)	0.8162 (0.4625–1.4402)	0.4833
No	209	37 (17.70)	172 (82.30)		
History of hypertension					
Yes	64	11 (17.19)	53 (82.81)	1.0657 (0.5177–2.1937)	0.8629
No	282	46 (16.31)	236 (83.69)		
History of diabetes mellitus					
Yes	91	14 (15.38)	77 (84.62)	0.9134 (0.4727–1.7651)	0.7876
No	253	42 (16.60)	211 (83.40)		
History of obesity					
Yes	61	10 (16.39)	51 (83.61)	1.0145 (0.4803–2.1429)	0.9698
No	284	46 (16.20)	238 (83.80)		
High systolic pressure		55	308		
Age (years)					
≤ 20	22	2 (9.09)	20 (90.91)	[6.1702]	0.1868
[20–35]	72	7 (9.72)	65 (90.28)		
[35–50]	101	14 (13.86)	87 (72.28)		
[50–65]	122	26 (21.31)	96 (78.69)		
> 65	46	6 (13.04)	40 (86.96)		
Age ≤ 20					
Yes	22	2 (9.09)	20 (90.91)	1.8403 (0.4178–8.1066)	0.4201
No	341	53 (15.54)	288 (84.46)		
Gender					
Female	188	27 (14.36)	161 (85.64)	1.1358 (0.6398–2.0164)	0.6637
Male	175	28 (16.00)	147 (84.00)		
Income level					
Low	167	27 (16.17)	140 (83.83)	[0.8966]	0.6387
Middle	192	28 (14.58)	164 (85.42)		
High	4	0 (0.00)	4 (100.00)		
Low income level					
Yes	152	25 (16.45)	127 (83.55)	0.8148 (0.4626–1.4352)	0.4784
No	196	28 (14.29)	168 (85.71)		
Smoking					
Yes	20	4 (20.00)	16 (80.00)	1.4321 (0.4602–4.4561)	0.5352
No	343	51 (14.87)	292 (85.13)		
Alcohol consumption					
Yes	85	14 (16.47)	71 (83.53)	1.1398 (0.5879–2.2100)	0.6985
No	278	41 (14.75)	237 (85.25)		
Physical activity					
Yes	154	25 (16.23)	129 (83.77)	1.1563 (0.6494–2.0591)	0.6217
No	209	30 (14.35)	179 (85.65)		
History of hypertension					
Yes	64	12 (18.75)	52 (81.25)	1.4383 (0.7051–2.9339)	0.3177
No	282	39 (13.83)	243 (86.17)		
History of diabetes mellitus					
Yes	91	16 (17.58)	75 (82.42)	1.4225 (0.7411–2.7303)	0.2895
No	253	33 (13.04)	220 (86.96)		
History of obesity					
Yes	61	8 (13.11)	53 (86.89)	0.8946 (0.3965–2.0185)	0.7885
No	284	41 (14.44)	243 (85.56)		
High blood pressure		47	316		
Age (years)					
≤ 20	22	2 (9.09)	20 (90.91)	[8.3676]	0.0790
[20–35]	72	4 (5.56)	68 (94.44)		
[35–50]	101	14 (13.86)	87 (86.14)		
[50–65]	122	23 (18.85)	99 (81.15)		
> 65	46	4 (8.70)	42 (91.30)		
Age ≤ 20					
Yes	22	2 (9.09)	20 (90.91)	1.5184 (0.3435–6.7126)	0.5818
No	341	45 (13.20)	296 (86.80)		
Gender					
Female	188	23 (12.23)	165 (87.77)	1.1402 (0.6177–2.1049)	0.6748
Male	175	24 (13.71)	151 (86.29)		
Income level					
Low	167	23 (13.77)	144 (86.23)	[0.7299]	0.6942
Middle	192	24 (12.5)	168 (87.5)		
High	4	0 (0.00)	4 (100.00)		
Low income level					
Yes	152	21 (13.82)	131 (86.18)	0.8372 (0.4568–1.5345)	0.5654
No	196	24 (12.24)	172 (87.76)		
Smoking					
Yes	20	3 (15.00)	17 (85.00)	1.1992 (0.3376–4.2597)	0.7787
No	343	44 (12.83)	299 (87.17)		
Alcohol consumption					
Yes	85	12 (14.12)	73 (85.88)	1.1413 (0.5634–2.3119)	0.7136
No	278	35 (12.59)	243 (87.41)		
Physical activity					
Yes	154	22 (14.29)	132 (85.71)	1.2267 (0.6632–2.2690)	0.5150
No	209	25 (11.96)	184 (88.04)		
History of hypertension					
Yes	64	11 (17.19)	53 (82.81)	1.5147 (0.7215–3.1799)	0.2725
No	282	34 (12.06)	248 (87.94)		
History of diabetes mellitus					
Yes	91	13 (14.29)	78 (85.71**)**	1.1936 (0.5944–2.3966)	0.6188
No	253	31 (12.25)	222 (87.75)		
History of obesity					
Yes	61	6 (9.84)	55 (90.16)	0.7284 (0.2930–1.8109)	0.4953
No	284	37 (13.03)	247 (86.97)		
High heart rate		48	315		
Age (years)					
≤ 20	22	3 (13.64)	19 (86.36)	[1.7282]	0.7856
[20–35]	72	8 (11.11)	64 (88.89)		
[35–50]	101	14 (13.86)	87 (86.14)		
[50–65]	122	19 (15.57)	103 (84.43)		
> 65	46	4 (8.7)	42 (91.30)		
Age ≤ 20					
Yes	22	3 (13.64)	19 (86.36)	0.9624 (0.2738–3.3833)	0.9523
No	341	45 (13.20)	296 (86.80)		
Gender					
Female	188	28 (14.89)	160 (85.11)	0.7373 (0.3987–1.3636)	0.3314
Male	175	20 (11.43)	155 (88.57)		
Income level					
Low	167	22 (13.17)	145 (86.83)	[0.6269]	0.7309
Middle	192	26 (13.54)	166 (86.46)		
High	4	0 (0.00)	4 (100.00)		
Low income level					
Yes	152	19 (12.50)	133 (87.50)	1.0133 (0.5530–1.8566)	0.9660
No	196	26 13.27)	170 (86.73)		
Smoking					
Yes	20	1 (5.00)	19 (95.00)	0.3318 (0.0434–2.5349)	0.2876
No	343	47 (13.70)	296 (86.30)		
Alcohol consumption					
Yes	85	10 (11.76)	75 (88.24)	0.8421 (0.4005–1.7708)	0.6505
No	278	38 (13.67)	240 (86.33)		
Physical activity					
Yes	154	19 (12.34)	135 (87.66)	0.8736 (0.4699–1.6239)	0.6692
No	209	29 (13.88)	180 (86.12)		
History of hypertension					
Yes	64	9 (14.06)	55 (85.94)	1.0851 (0.4951–2.3781)	0.8383
No	282	37 (13.12)	245 (86.88)		
History of diabetes mellitus					
Yes	91	10 (10.99)	81 (89.01)	0.7690 (0.3641–1.6241)	0.4911
No	253	35 (13.83)	218 (86.17)		
History of obesity					
Yes	61	14 (22.95)	47 (77.05)	2.5221 (1.2442–5.1124)	0.0103*
No	284	30 (10.56)	254 (89.44)		

N, n: Number; (95% CI): 95% confidence intervals; OR: Odd ratio; Bold value with *: Significant., Chi-square value are in []

The mean value of the body mass index was 24.79 ± 4.03 kg/m^2^ (16.30–37.6 kg/m^2^) in our sample population. Approximately forty percent (39.94%) of our sample population was with high body mass index, 31.12% for overweight and 8.81% for obesity. Examining high BMI with respect to sociodemographics, lifestyle and medical history, participants in age group above 20 years were significantly more prone to overweight/obesity than younger ones (OR: 7.2221 (1.6616–31.3910), p = 0.0084), (Table 4).

**Table 4:** Distribution of high BMI according to socio-economic, lifestyle and medical history parameters

Variable	N	Present n (%)	Absent n (%)	OR (95% CI)	p value
High BMI		145	218		
Age (years)					
≤ 20	22	2 (9.09)	20 (90.91)	[19.2034]	0.0007*
[20–35]	72	19 (26.39)	53 (73.61)		
[35–50]	101	46 (45.54)	55 (54.46)		
[50–65]	122	59 (48.36)	63 (51.64)		
> 65	46	19 (41.30)	27 (58.70)		
Age ≤ 20					
Yes	22	2 (9.09)	20 (90.91)	7.2221 (1.6616–31.3910)	0.0084*
No	341	143 (41.94)	198 (58.06)		
Gender					
Female	188	81 (43.09)	107 (56.91)	0.7617 (0.4995–1.1613)	0.2059
Male	175	64 (36.57)	111 (63.43)		
Income level					
Low	167	59 (35.33)	108 (64.67)	[2.8105]	0.2453
Middle	192	84 (43.75)	108 (56.25)		
High	4	2 (50.00)	2 (50.00)		
Low income level					
Yes	152	57 (37.50)	95 (62.50)	1.2956 (0.8558–1.9616)	0.2210
No	196	86 (43.88)	110 (56.12)		
Smoking					
Yes	20	4 (20.00)	16 (80.00)	0.3582 (0.1173–1.0939)	0.0715
No	343	141 (41.11)	202 (58.89)		
Alcohol consumption					
Yes	85	38 (44.71)	47 (55.29)	1.2921 (0.7907–2.1115)	0.3064
No	278	107 (38.49)	171 (61.51)		
Physical activity					
Yes	154	57 (37.01)	97 (62.99)	0.8080 (0.5271–1.2385)	0.3279
No	209	88 (42.11)	121 (57.89)		
History of hypertension					
Yes	64	29 (45.31)	35 (54.69)	1.3151 (0.7607–2.2735)	0.3268
No	282	109 (38.65)	173 (61.35)		
History of diabetes					
Yes	91	36 (39.56)	55 (60.44)	1.0015 (0.6135–1.6346)	0.9954
No	253	100 (39.53)	153 (60.47)		
History of obesity					
Yes	61	29 (47.54)	32 (52.46)	1.4335 (0.8218–2.5005)	0.2045
No	284	110 (38.73)	174 (61.27)		

N, n: Number; BMI: Body mass index; (95% CI): 95% confidence intervals; OR: Odd ratio; Bold value with *: Significant, Chi-square value are in []

### *H. pylori* infection in the study population

The prevalence of *H. pylori* in the study population was 65.94% (239/363); 63.83% (120/188) among women and 68.00% (119/175) among men, but the difference related to the gender was not significant (p = 0.4025). Rate of *H. pylori* infection with age was relatively constant in each age group (65.56 to 77.27%) except that of the group in the age range 20–35 years which was slightly low (54.16%), but with no significant difference (X^2^ = 6.8206, p = 0.1457). Participants from the low and middle income groups were most affected by *H. pylori* infection compared to those from the high income group, but the different was not significant (X^2^ = 3.4654, p = 0.1768). Regarding lifestyle of participants, 65.00% and 64.71% of smokers and alcohol consumers versus 35.00% and 35.29% were *H. pylori* infected. However, the differences were not significant (p = 0.935 and 0.801 respectively), (Table 5).

**Table 5:** *H. pylori* infection in the study population and potentials risk factors for infection

Variables	Number (%)	HP +ve, n = 239	HP −ve, n = 124	X^2^ (p value)
Sex				
Female	188 (51.79)	120 (63.83)	68 (36.17)	0.7008 (0.402)
Male	175 (48.21)	119 (68.00)	56 (32.00)	
Age (years)				
≤ 20	22 (6.06)	17 (77.27)	5 (22.73)	6.8206 (0.1457)
[20–35]	72 (19.83)	39 (54.16)	33 (45.84)	
[35–50]	101 (27.82)	71 (70.30)	30 (29.70)	
[50–65]	122 (33.61)	80 (65.56)	42 (34.44)	
> 65	46 (12.67)	32 (69.56)	14 (30.44)	
Income level ($/month)				
Low class (≤ 2500)	152 (41.87)	104 (68.42)	48 (31.58)	3.4654 (0.1768)
Middle class (2500–8500)	192 (52.89)	125 (65.10)	67 (34.90)	
Elite (≥ 8500)	4 (1.10)	1 (25.00)	3 (75.00)	
Smoking				
Yes	20 (5.51)	13 (65.00)	7 (35.00)	0.0066 (0.935)
No	343 (94.49)	226 (65.89)	117 (34.11)	
Alcohol consumption				
Yes	85 (23.42)	55 (64.71)	30 (35.29)	0.0635 (0.801)
No	278 (76.58)	184 (66.19)	94 (33.81)	

N: Number; HP: *H. pylori*; +ve: Positive; −ve: Negative; X^2^: Chi-square

### *H. pylori* infection and dyslipidemia

Higher prevalence of each dyslipidemia marker in abnormal range was found among *H. pylori* positive individuals compared to *H. pylori* negative ones, with a significant difference regarding total cholesterol and LDL cholesterol levels (p = 0.0007 and 0.0024 respectively). In fact, 73.21% versus 26.79%, 77.04 versus 22.96%, 69.72 versus 30.28%, and 68.75 versus 31.25% of *H. pylori* positive subjects versus *H. pylori* negative ones were with hypercholesterolemia, high LDL, low HDL and high triglycerides respectively (Table 6).

**Table 6:** *Helicobacter pylori* infection and risk markers of cardiovascular diseases

Variable	Number (%)	HP +ve n (%)	HP −ve n (%)	[t value; p-value] (X^2^ value; p-value)
Total cholesterol (g/l), Mean ± SD, (Range)	215.38 ± 59.58, (86–412)	223.2 ± 3.781	200.2 ± 5.096	[3.562; 0.0004*]
Hypo cholesterol (< 150)	59 (16.25)	30 (50.85)	29 (49.15)	(14.58; 0.0007*)
Normal (150 to 200)	80 (22.03)	45 (56.25)	35 (43.75)	
Hyper cholesterol (> 200)	224 (61.70)	164 (73.21)	60 (26.79)	
LDL (g/l), Mean ± SD, (Range)	110.02 ± 45.21,(12.98 229.6)	116.6 ± 2.954	97.26 ± 3.734	[3.459; 0.0006*]
Hypo (< 90)	130 (35.52)	76 (58.46)	54 (41.54)	(12.056; 0.0024*)
Normal (90 to 130)	98 (26.99)	59 (60.20)	39 (39.80)	
Hyper (> 130)	135 (37.19)	104 (77.04)	31(22.96)	
HDL (g/l), Mean ± SD, (Range)	51.79 ± 21.03, (4.3–119)	51.01 ± 1.366	52.92 ± 1.912	[0.8027; 0.4227]
Hypo (< 40)	142 (39.12)	99 (69.72)	43 (30.28)	(4.966; 0.0831)
Normal (40 to 60)	185 (50.96)	122 (65.94)	63 (34.06)	
Hyper (> 60)	36 (9.97)	18 (50.00)	18 ()	
TGA (g/l), Mean ± SD, (Range)	145.91 ± 61.94, (13.39–318)	146.2 ± 3.513	142.5 ± 4.964	[0.6168; 0.5378]
Hypo (< 40)	6 (1.65)	3 (50.00)	3 (50.00)	(1.5816; 0.4535)
Normal (40–150)	197 (54.26)	126 (63.96)	71 (36.04)	
Hyper (> 150)	160 (44.07)	110 (68.75)	50 (31.25)	
BMI (kg/m^2^), Mean ± SD, (Range)	24.79 ± 4.03, (16.30–37.6)	24.84 ± 0.2515	24.7 ± 0.3862	[0.3146; 0.7609])
Normal (18.5–24.9)	218 (60.05)	142 (65.14)	76 (34.86)	(0.1493; 0.9281)
Overweight (25–29.9)	113 (31.12)	76 (67.26)	37 (32.74)	
Obesity (≥ 30)	32 (8.81)	21 (65.62)	11 (34.38)	
DBP (mmHg), Mean ± SD, (Range)	77.024 ± 11.21 (41–104)	77.18 ± 0.7757	77.35 ± 0.925	[0.1324; 0.8947]
Hypo (< 80)	225 (61.98)	146 (68.89)	79 (31.11)	(1.10; 0.576)
Normal (80 to 90)	78 (21.48)	50 (64.10)	28 (35.09)	
Hyper (> 90)	60 (16.52)	43 (71.67)	17 (28.33)	
SBP (mmHg), Mean ± SD, (Range)	124.11 ± 19.29 (80–191)	121.7 ± 1.284	137 ± 12.3	[1.732; 0.0841]
Hypo (< 120)	163 (44.90)	114 (69.94)	49 (30.06)	(2.2252; 0.3287)
Normal (120 to 140)	145 (39.94)	91 (62.76)	54 (37.24)	
Hyper (> 140)	55 (15.15)	34 (61.82)	21 (38.18)	
Blood pressure (mmHg)	/, (41/80–104/191)	/	/	/
Hypo (< 120/80)	152 (41.87)	98 (64.47)	54 (35.53)	(0.2373; 0.8881)
Normal (120–140/80–90)	164 (45.17)	110 (97.07)	54 (2.93)	
Hyper (> 140/90)	47 (12.94)	31 (65.96)	16 (34.04)	
Heart rate (beat/min)	79.53 ± 12.10 (45–114)	80.00 ± 0.8606	78.56 ± 1.12	[0.9968; 0.3195]
Normal (60 to 100)	287 (79.06)	186 (64.81)	101 (35.19)	(1.235; 0.5394)
Tachycardia (> 100)	48 (13.22)	35 (72.92)	13 (27.08)	
Bradycardia (< 60)	28 (7.71)	18 (64.28)	10 (35.72)	

HP: *H. pylori*; +ve: Positive; −ve: Negative; DBP: Diastolic blood pressure; SBP: Systolic blood pressure; SD: standard deviation; X^2^: Chi-square; Bold value with *: Significant

In addition, *H. pylori* infected individuals compared to *H pylori* uninfected ones had a significantly higher mean value of total cholesterol concentration (223.2 ± 3.781 versus 200.2 ± 5.096 g/l, t = 3.562, p = 0.0004) and LDL concentration (116.6 ± 2.954 versus 97.26 ± 3.734 g/l, t = 3.459, p = 0.0006) and a marginal lower value of HDL (51.01 ± 1.366 versus 52.92 ± 1.912 g/l, t = 0.8027, p = 0.4227) and higher triglycerides concentration (146.2 ± 3.513 versus 142.5 ± 4.964, t = 0.6168, p = 0.5378), (Table 6).

The strength of the association of *H. pylori* infection and each dyslipidemia’s marker were analyzed by determining the OR and the corresponding 95% CI value. Our results show that compared to *H. pylori* negative patients, the OR of *H. pylori* status on the prevalence of hypercholesterolemia, high LDL, low HDL and high triglycerides were 2.3324 (95% CI 1.4935–3.6423, p = 0.0002), 2.3096 (95% CI 1.4289–3.7331, p = 0.0006), 1.3321(95% CI 0.8489–2.0902, p = 0.2123) and 1.2620 (95% CI 0.8129–1.9592, p = 0.2327) respectively. We also adjusted for confounding factors (age, sex, income in model 1; smoking, alcohol consumption, physical activity in model 2; family histories of hypertension, diabetes mellitus and obesity in model 3 and the overall confounders in model 4) in the logistic regression and a similar results was noticed (OR: 2.5944 (95% CI 1.5766–4.2692, p = 0.0002; OR: 2.6794 (95% CI 1.5839–4.5328, p = 0.0002; OR: 1.4103 (95% CI 0.8588–2.3160, p = 0.1743; and OR: 1.1116 (95% CI 0.6918–1.7861, p = 0.6620 respectively in model 4), (Table 7).

**Table 7:** Relationship between *Helicobacter pylori* infection and dyslipidaemia, heart dysfunction, overweight/obesity adjusting with potential confounders (socio-economic lifestyle and medical history parameters) assessed by multivariable linear regression analysis

Variable	Univariate logistic regression		Multivariate logistic regression							
	OR (95% CI)	p value	Model 1		Model 2		Model 3		Model 4	
			OR (95% CI)	p value	OR (95% CI)	p value	OR (95% CI)	p value	OR (95% CI)	p value
Dyslipidemia										
Hyper total cholesterol	2.3324 (1.4935–3.6423)	0.0002*	2.5848 (1.6235–4.1155)	0.0001*	2.4100 (1.5324–3.7903)	0.0001*	2.3373 (1.4610–3.7391)	0.0004*	2.5944 (1.5766–4.2692)	0.0002*
High LDL-C	2.3096 (1.4289–3.7331)	0.0006*	2.5793 (1.5560–4.2754)	0.0002*	2.3600 (1.4550–3.8280)	0.0005*	2.3612 (1.4412–3.8684)	0.0006*	2.6794 (1.5839–4.5328)	0.0002*
Low HDL-C	1.3321 (0.8489–2.0902)	0.2123	1.2658 (0.7919–2.0234)	0.3247	1.3480 (0.8565–2.1216)	0.1969	1.4080 (0.8786–2.2563)	0.1550	1.4103 (0.8588–2.3160)	0.1743
High TG	1.2620 (0.8129–1.9592)	0.2327	1.1407 (0.7249–1.7951)	0.5693	1.2714 (0.8154–1.9825)	0.2894	1.2431 (0.7885–1.9598)	0.3487	1.1116 (0.6918–1.7861)	0.6620
Heart dysfunction										
High DBP	1.3807 (0.7510–2.5384)	0.2991	1.3082 (0.7348–2.3291)	0.3614	1.4359 (0.7764–2.6554)	0.2488	1.4466 (0.7600–2.7534)	0.2609	1.6772 (0.8404–3.3474)	0.1424
High SBP	0.8135 (0.4495–1.4723)	0.4952	0.8575 (0.4655–1.5796)	0.6218	0.8020 (0.4412–1.4578)	0.4692	0.9498 (0.4999–1.8047)	0.8751	0.9589 (0.4900–1.8764)	0.9024
Hypertension	1.0060 (0.5270–1.9204)	0.9855	1.0781 (0.5527–2.1032)	0.8253	0.9874 (0.5151–1.8927)	0.9695	1.0980 (0.5540–2.1760)	0.7888	1.0866 (0.5292–2.2310)	0.8210
High heart rate	1.4646 (0.7441–2.8829)	0.2694	1.3885 (0.6947–2.7753)	0.3529	1.4854 (0.7514–2.9366)	0.2551	1.4730 (0.7065–3.0708)	0.3015	1.3765 (0.6460–2.9330)	0.4077
BMI										
Overweigh/Obesity	1.0813 (0.6936–1.6859)	0.7300	1.1474 (0.7240–1.8185)	0.5585	1.1063 (0.7054–1.7349)	0.6601	1.1104 (0.6992–1.7635)	0.6571	1.1785 (0.7234–1.9201)	0.5095

DBP: Diastolic blood pressure; SBP: Systolic blood pressure; N, n: Number, (95% CI): 95% confidence intervals; OR: Odd ratio. Bold value with *: Significant. Model 1: Adjusted with sociodemographic and economic factors (sex, gender and income level); Model 2: Adjusted with lifestyle factors (smoking, alcohol consumption, physical activity); Model 3: Adjusted with medical history (history of hypertension, diabetes mellitus, and obesity); Model 4: Adjusted for all the confounding factors (Sociodemographic and economic, lifestyle and medical history)

### *H. pylori* infection and heart dysfunction

Regarding heart dysfunction, high diastolic blood pressure, high systolic blood pressure and hypertension were commonly found in *H. pylori* infected individuals compared to non-infected ones. But the difference was not significant (X^2^: 1.10, p = 0.576; X^2^: 2.2252, p = 0.3287; X^2^: 0.0003, p = 0.9855 respectively). Also, higher heart rate was frequent among infected individuals compared to uninfected ones, but with a non-significant difference (X^2^: 1.235, p = 0.5394).

Concerning the mean value of heart dysfunction markers, a similar mean value of diastolic pressure (77.18 ± 0.7757 versus 77.35 ± 0.925 mmHg), a lower mean value of systolic pressure (121.7 ± 1.284 versus 137 ± 12.3 mmHg) and a higher mean heart rate value (80.00 ± 0.8606 versus 78.56 ± 1.12 beat/min) were noticed in *H. pylori* infected patients compared to non-infected ones, but with non-significant differences (t = 0.1324, p = 0.8947; t = 1.732, p = 0.0841; t = 0.9968, p = 0.3195 respectively), (Table 6).

The strength of the association between *H. pylori* infection and heart dysfunction, shows an OR of 1.3807 (95% CI 0.7510–2.5384, p = 0.2991), 1.0060 (95% CI 0.5270–1.9204, p = 0.9855) and 1.4646 (95% CI 0.7441–2.8829, p = 0.2694) for diastolic pressure, hypertension and high heart rate respectively among *H. pylori* infected patients compared with uninfected ones, while that of systolic pressure was 0.8135 (95% CI 0.4495–1.4723, p = 0.4952). This relation persist even when adjusted with confounders in model 1, 2, 3 and 4) with the OR of 1.6772 (0.8404–3.3474, p = 0.1424); 1.0866 (0.5292–2.2310, p = 0.8210); 1.3765 (0.6460–2.9330, p = 0.4077) and 0.9589 (0.4900–1.8764, p = 0.9024) respectively regarding model 4, (Table 7).

### *H. pylori* and overweigh/obesity

High body mass index were commonly found among *H. pylori* positive subjects. In fact, 67.26% versus 32.74% of individuals with overweight and 65.62% versus 34.38% of obese individuals were *H. pylori* infected. However the difference was non-significant compared to subjects with normal weight (X^2^: 0.1493, p = 0.9281). Regarding BMI mean value according to *H. pylori* status, a slightly higher and non-significant BMI mean value was recorded among *H. pylori* infected individuals compared with non-infected ones (24.84 ± 0.2515 versus 24.7 ± 0.3862 kg/m^2^, t = 0.3146, p = 0.7609), (Table 6).

The strength of the association between *H. pylori* infection and high BMI was analyzed by determining the OR and the corresponding 95% IC value. Our results show that compared to *H. pylori* negative patients, the crude OR of *H. pylori* status on the prevalence of high BMI was 1.0813 (95% CI 0.6936–1.6859, p = 0.7300) and the adjusted OR for confounding factors in model 4 using the logistic regression was 1.1785 (95% CI 0.7234–1.9201, p = 0.5095), (Table 7).

## Discussion

In this cross-sectional study of dyspeptic patients who underwent upper endoscopy with *H. pylori* detection in three health facilities in Cameroon, we investigated the association of *H. pylori* infection and risk factors for cardiovascular disease (that is dyslipidemia, high blood pressure and heart rate, high body mass index). We also evaluated the impact of potential confounders such socio-economic parameters, lifestyle and medical histories on this association.

Our results showed that *H. pylori* infected individuals were more at risk of hypercholesterolemia, high LDL cholesterol, low HDL cholesterol and high triglycerides levels, with a significant difference regarding the proportion of infected subjects with hypercholesterolemia (p = 0.0002) or high LDL-cholesterol (p = 0.0006), and the mean value of total cholesterol (p = 0.0004) or LDL cholesterol levels (p = 0.0006) compared to non-infected ones. This positive relationship between *H. pylori* infection and markers of dyslipidemia persisted even after adjusted with confounders, indicating *H. pylori* infection as a risk factor for dyslipidemia.

Our result is consistent to that of studies investigating on the impact of *H. pylori* colonization on lipid profile. In fact, *H. pylori* infection was significantly associated with higher total cholesterol level (p < 0.001), higher LDL cholesterol level (p < 0.001), and lower HDL cholesterol level (p < 0.001) in a cross-sectional study of 37,263 asymptomatic subjects [35]. Similarly, a lower HDL-cholesterol and higher triglyceride levels among CLO-positive patients was reported [36]. In a study comparing the lipid profiles of *H. pylori* uninfected patients without CVD to *H. pylori* infected patients with CVD, the authors demonstrate no significant changes in triglycerides, total cholesterol, and LDL levels but a significant lower HDL level in both groups [37]. Regarding *H. pylori* eradication on the lipid profile, a rise in HDL levels and a drop in low density lipoprotein (LDL) levels was noticed with *H. pylori* eradication [38], a significant decreased of the total cholesterol (TC) and LDL-C levels in *H. pylori* infected patients after receiving anti-*H. pylori* treatment [39]. These previous findings, coupled with ours show that *H. pylori* infection promotes dyslipidemia by raising cholesterol, LDL cholesterol and triglyceride levels and reducing HDL cholesterol level.

Inflammatory cytokines such as tumor necrosis factor-α (TNF-α), interleukin-1, interleukin-6 production is stimulated in the host by *Helicobacter pylori’* lipopolysaccharides [40]. This up regulate of inflammatory cytokines induces by *H. pylori* infection impairs lipids metabolism [41, 42], which lead to atherosclerosis in *H. pylori* infected patients [43, 44]. Some authors reported that increasing in serum levels of some pro-inflammatory cytokines (IL-1b, IL-8, and TNF-α) goes along with the increment of cardiovascular diseases risk [45]. In fact, lipoprotein lipase activity is inhibited by TNF-α of [46], in such condition lipids are transferred from the tissue which turn up the blood level of triglycerides and reduced that of HDL cholesterol [47, 48]. IL-6 and TNF-α also interfere in lipid metabolism by increasing liver cholesterol synthesis. In fact, these cytokines impair the 3-hydroxy-3-methyl glutaryl coenzyme A reductase gene expression and reduce liver cholesterol catabolism by inhibiting cholesterol hydroxylase [49]. Hence, *H. pylori* infection may has a role in promoting atherosclerosis through dyslipidemia since it had been shown that changes in cholesterol levels are associated with changes in CVD incidence rate [50]. It is demonstrated that, a 10% move to lower position in serum cholesterol produces a fall in CHD risk of 50% in 40 years aged individuals, 40%, 30% and 20% in those aged 50, 60 and 70 years old [50]. Regarding HDL cholesterol, a 1 mg/dl rise in HDL level is associated with a 2% and 3% drop in coronary risk in men and women respectively [50]. Since both LDL cholesterol and HDL cholesterol are directly associated with atherosclerosis and CVD, they are principal marker for high blood cholesterol therapy [50]. Thus, *H. pylori* eradication is beneficial to reduce the occurrence of dyslipidemia, thereby preventing the occurrence of cardiovascular disease.

However, there are contradictory results indicating that *H. pylori* infection may not alter lipid profile or interfere in lipid metabolism. A non-significant difference between plasma cholesterol, triglyceride levels or lipid profile and *H. pylori* seropositivity was reported by Rengström et al. [51] and Jamshid et al. [6] in their study population. Patel et al. also did not find differences in the plasma cholesterol or triglyceride levels, among *H. pylori* positive and negative patients [52]. The difference in the methods used for *H. pylori* detection, the sample size, the type of population as well as the potential confounders choose for analysis may explain such discrepancy among findings. In fact, serological tests characterized by their inability to differentiate between current and past infection, since the antibody titers can remain high for months after the elimination of the infection and also linked to the cross reactivity with other antibodies are widely used for *H. pylori* detection in studies related *H. pylori* infection and CVD [35]. Hence, these studies are not reliable to check for the relationship between *H. pylori* current infection and CVD.

The impact of *H. pylori* infection was also evaluation on markers of heart dysfunction; high blood pressure (hypertension) and high heart rate known as cardiovascular risk factors. High blood pressure increases the amount of work for heart. This heart extra effort extends the size of heart muscle which become inflexible, causing heart dysfunction. A two–threefold increase incidence of atherosclerotic CVD occurs along with hypertension [50]. Even individuals with high normal blood pressure (BP) values are in high risk to develop CVD [50]. Hypertension favors the development of all clinical manifestations of CHD such as myocardial infarction, angina pectoris, and sudden death. Hypertension is also implicated in the occurrence of atherothrombotic brain infarction such as intracerebral hemorrhage [50]. In 40–70 years aged individuals, every 20-point or 10-point increase in systolic blood pressure or diastolic blood pressure respectively starting at 115/75 mmHg, rise the risk of heart attack and folded over once stroke [50].

Our results on heart dysfunction related to *H. pylori* status showed that *H. pylori* infected individuals were more times subjected to a higher blood pressure (hypertension), to a higher diastolic blood pressure and to a higher heart rate compared with uninfected ones, but the differences were non-significant. We also saw that *H. pylori* infected individuals were not commonly found among subjects with a higher systolic blood pressure. This positive relationship between *H. pylori* infection and hypertension, high diastolic pressure and high heart rate although non-significant persist even after adjusted with confounders in each model type, indicating that *H. pylori* infection is related to these heart functional abnormalities.

Our finding is consistent with previous study investigating the effect of *H. pylori* on blood pressure. Hartog et al. in their study found that *H. pylori* infection was weakly associated with hypertension with a non-significant difference [53]. Xiong et al., found a significantly high blood pressure with a higher diastolic blood pressure in *H. pylori*-positive adults compared to for *H. pylori* negative but not to systolic blood pressure [54]. Similarly, Wan et al. in their cross-sectional study noticed a positive link between *H. pylori* infection with diastolic blood pressure but not with systolic blood pressure [55]. A community-based study in Iran also found that *H. pylori* immunoglobulin G (IgG) antibodies are significantly associated with hypertension [56], while Migneco et al. noticed a significant decrease in diastolic blood pressure after *H. pylori* eradication in hypertensive patients [57].

Controversial data showing that *H. pylori* infection did not influence neither systolic blood pressure nor diastolic blood pressure have been published in the literature [58, 59]. A cross-sectional study on hypertensive and normotensive Chinese Mongolian subjects did not find a link between *H. pylori* and hypertension [60]. The studied population, sample size, methods for *H. pylori* detection and adjustment for potential confounders may explain such inconsistent findings.

The mechanisms sustained the link between *H. pylori* infection and hypertension have been documented despite the contradictory results regarding this issue. *H. pylori* colonization impaired digestion and absorption of nutrients such as vitamin B6, vitamin B12, and folic acid in the stomach [61] resulting in the deficiency in these important coenzymes for methionine metabolism and turn up homocysteine levels among infected individuals [62]. Homocysteine causes platelet aggregation and vasoconstriction by inhibiting nitric oxide (NO) secretion in endothelial cells, it also favors the binding of lipoproteins to fibrinogen which predisposes to arteriosclerosis and hypertension [63]. Such reasons may explain why *H. pylori* infection are more subjected to high blood pressure than uninfected ones. *H. pylori* infected subjects also had increased levels of fibrinogen, a marker of vascular inflammation. It inhibits the reduction of NO which result in vasoconstriction and hypertension in peripheral blood vessel [64], this may justify the fact that individuals with *H. pylori* infection had high diastolic blood pressure but not high systolic blood pressure, since diastolic blood pressure widely relies to peripheral resistance. The dyslipidemia and dysglycemia observed among our population may also explain such high diastolic blood pressure, since it is documented that diastolic function is correlated with lipid profile [65].

Obesity represents a major public health problem regarding the increase number of affected individuals over the past century. An estimate rise of overweight/obesity of 27.55 and 47.1% respectively among adults and children has been reported between 1980 and 2013 [66], with the highest frequencies recorded among individuals from developed countries. In addition, obesity decreases the expected length of life and induces failure to perform function properly in many organs and systems [67]. Obesity is correlated to mortality due to chronic diseases such as diabetes, chronic kidney disease, gastrointestinal disease, and cardiovascular disease [68].

In the current study, 39.94% of the subjects were with high body mass index, 31.12% for overweight and 8.81% for obesity. Regarding overweigh/obesity related to *H. pylori* status, our findings showed that the majority of individuals with overweight and obesity were *H. pylori* infected, with the OR higher than one, but the differences were not significant.

Our finding is in accordance with those of previous studies demonstrating that *H. pylori* positive subjects are more in risk of having high BMI than *H. pylori* negative ones or showing an association between *H. pylori* infection and overweight/obesity [10]. A significant association between obesity and *H. pylori* positivity was noticed among a young obese group of Turks compare to the control group [69], in Czech people [70] and in Swedish subjects [71]. A similar positive correlation between *H. pylori* infection and increased BMI or overweight/obesity was noticed in an Israel cohort of 235,107 individuals [72], and in a systematic review among Chinese population [73]. Moreover, *H. pylori* infection rates of 37.36%, 41.88%, 45.77% (p for trend = 0.006) were recorded respectively among normal, overweight and obese subjects, indicating trend of increasing of BMI with *H. pylori* infection [74].

There are conflicted findings reporting no link between *H. pylori* infection and overweight/obesity in the literature, despite the large documentation on the positive association between this comorbidities. In fact, *H. pylori* colonization was found to be not related with overweight/obesity in Japanese and Greek subjects [75–77]. A similar negative relationship was noticed in studies from USA [78, 79].

Suggestions on the mechanisms that might link *H. pylori* infection to high BMI have been illustrated. One mechanism is related to leptin and ghrelin, gastrointestinal hormones involved in energy balance. Ghrelin causes excessive eating and obesity by stimulating food intake [80], whereas leptin reduces excessive eating and obesity by inhibiting eating [81]. Lower serum leptin and ghrelin levels has been demonstrated in *H. pylori*-positive patients [82]. Another mechanism is related to insulin resistance. Insulin resistance is known as an important risk factor for metabolic disorders such as obesity [83]. It has been shown that *H. pylori* infection are more subjected to insulin resistance [84] and therefore may be more likely to get obesity than uninfected ones. The interaction between *H. pylori* invasion and obesity on the immune system has also incriminated as one mechanism. In fact, the aptitude of monocytes to convert into macrophages is reduced in obese patients [69], indicating an improvement of *H. pylori* survival in obese people’ immune environment. In other hands, intestinal immune system in the presence of microorganism invasion promotes the development of metabolic disease such as obesity [85]. In fact, to proliferate and differentiate into adipocytes, pre-adipocytes could develop phagocytic activity as macrophage-like cells [86], which suggest that the presence of *H. pylori* may stimulate the growth of adipose tissue and thereby obesity.

## Conclusion

Our finding showed that *H. pylori* infection is associated with increased risk of dyslipidemia in our sample population. In view of that, *H. pylori* eradication would be beneficial to reduce the occurrence of dyslipidemia, thereby preventing the occurrence of cardiovascular disease in our milieu. However, it would be more interested to follow these patients in order to assess the variation of lipid profile over the time in relation to *H. pylori* infection. Our further attention is focused on the pathophysiological pathways of *H. pylori* on lipid metabolic and on the assessment of *H. pylori* colonization as an independent risk factor for coronary heart disease, that is the pathogen might directly invade the vessel wall, leading to localized vascular inflammation and resulting in cardiovascular disease.

## Data Availability

The datasets used and/or analyzed during the current study are not publicly available because they are confidential but are available from the corresponding author on reasonable request.

## Abbreviations

CVD: Cardiovascular disease
CHD: Coronary heart disease
TC: Total cholesterol
LDL-C: Low density lipoprotein cholesterol
HDL-C: High density lipoprotein cholesterol
TG: Triglycerides
BP: Blood pressure
DBP: Diastolic blood pressure
SBP: Systolic blood pressure
BMI: Body mass index
TNF-α: Tumor necrosis factor-α
IL: Interleukin
HP or *H. pylori*: *Helicobacter pylori*
